# Transmigration of Mandibular Canines

**DOI:** 10.1155/2013/697671

**Published:** 2013-03-27

**Authors:** N. Umashree, Avinash Kumar, Tejavathi Nagaraj

**Affiliations:** ^1^Department of Oral Medicine and Maxillofacial Radiology, Al-Badar Dental College and Hospital, Gulbarga, Karnataka, India; ^2^Department of Orthodontics and Dentofacial Orthopedics, Al-Badar Dental College and Hospital, Gulbarga, Karnataka, India; ^3^Department of Oral Medicine and Maxillofacial Radiology, Sri Rajiv Gandhi College of Dental Sciences and Hospital, Karnataka, India

## Abstract

The purpose of this paper is to present the first case of unusual reverse oblique (110 degrees to midsagittal plane) migration of mandibular right canine crossing the jaw midline and piercing the lower border of the mandible at the level of the opposite canine and also to report two more cases of transmigrated mandibular canine and one case of transmigrating mandibular canine. Mandibular canines are “cornerstone” of dental arch; their importance is manifested by their efficiency in masticatory function, stability of dental arch, and aid in maintaining natural facial expression. Early detection of this anomaly can help preserving these canines by orthodontic intervention or by surgical transplantation. This developmental anomaly is properly diagnosed by radiographic evaluation, which is primarily based on the panoramic radiograph. In patients with overretained deciduous canines or missing permanent canines, an intraoral radiograph should be supplemented with panoramic radiograph. This paper discusses the importance of early diagnosis of canine transmigration in treatment planning and reviews the various possible treatment options.

## 1. Introduction

Migration of teeth is a well-documented ectopia, the mechanism that causes the migration of a tooth is still not clear [1]. An impacted tooth migrates to a location some distance away from the site of its development but usually remains within the same side of the arch [2]. Migration of a tooth across the jaw midline without the influence of any pathological entity is called transmigration [3]. The intraosseous migration of a tooth apparently starts during the early mixed dentition stage and may take place over a period of many years [4].

The incidence of transmigration has increased over the past 50 years with the introduction of dental panoramic tomography. Aydin et al. reported a panoramic radiographic survey of 4500 patients in a Turkish subpopulation, which revealed 14 cases of canine transmigration, out of which six were maxillary and eight mandibular canines, with an incidence of 0.31% [5]. Javid reported a radiographic survey of 1000 students which revealed only 1 case of transmigrated impacted mandibular canine [6]. Aktan and associates reported a panoramic radiographic survey of 5000 patients and observed that transmigration of impacted mandibular canines was greater than the maxillary canines. Women were affected more often than men [7]. Most of the cases reported in the literature were unilateral mandibular canine transmigration [3–6] and a very few were cases of bilateral canine transmigration [8, 9].

The aim of this paper is to report the first unusual case of reverse oblique migration of mandibular right canine crossing the jaw midline and piercing the lower border of the mandible which has never been reported in the literature so far. We also report additional two cases of transmigrated mandibular canine and one case of mandibular canine with a tendency to transmigrate. This paper also discusses the importance of early diagnosis in treatment planning and reviews the various possible treatment options for canine transmigration.

## 2. Case Reports

**Case 1**

**Figure 1:**
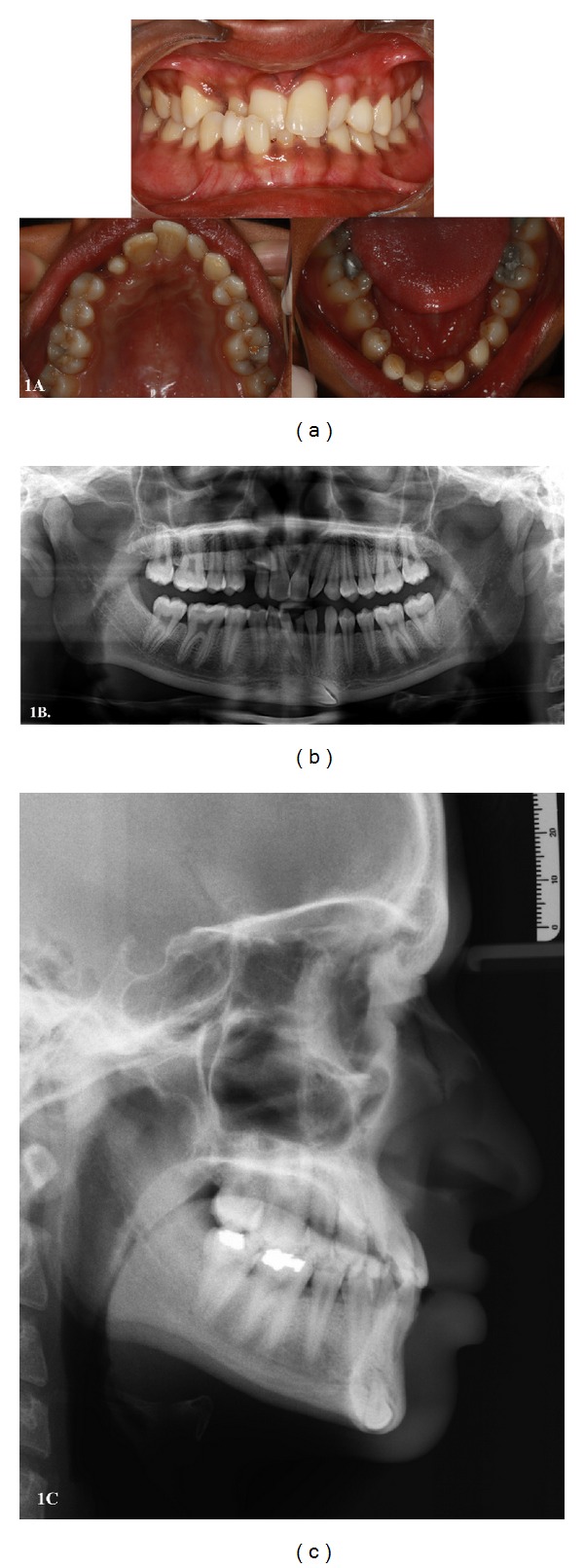
(a) Intraoral photographs showing clinically missing upper right canine, lower right canine and central incisors, and retained deciduous lower right canine and central incisor. (b) Panoramic radiograph showing impacted upper right canine and a reverse obliquely transmigrated lower right canine with its crown piercing the lower border of mandible at the level of opposite canine. (c) Lateral cephalogram showing obliquely lying lower right canine near the lower border of mandible.

A 20-year-old male presented with pain in front of the ear while opening and closing the mouth for one year. Intraoral examination revealed permanent maxillary right central and lateral incisor in cross-bite, peg-shaped maxillary lateral incisors, clinically missing permanent maxillary right canine, mandibular central incisors, and right canine, and retained deciduous mandibular right canine and left central incisor. TMJ examination revealed clicking and deviation of the jaw to right side while closing the mouth (Figure 1(a)). A panoramic radiograph was requested, which revealed impacted and ectopically erupting maxillary right canine, congenitally missing lower central incisors and reverse oblique (110° to midsagittal plane) transmigration of mandibular right canine crossing the jaw midline and piercing the lower border of the mandible and causing erosion of the inner (endosteal) cortex at the level of opposite canine. The radiographic position of the tooth was not in accordance with the classification given by Mupparapu [10] for mandibular canine transmigration (Figure 1(b)). The lateral cephalogram revealed obliquely lying permanent mandibular right canine near the lower border of mandible (Figure 1(c)). No pathologic finding was associated with the transmigrated tooth. Surgical removal of the canine was decided. However, the patient declined the surgical intervention as the tooth was asymptomatic. The patient was informed of the consequence of pathologic fracture and was scheduled for periodic radiographic monitoring.

**Case 2**

**Figure 2:**
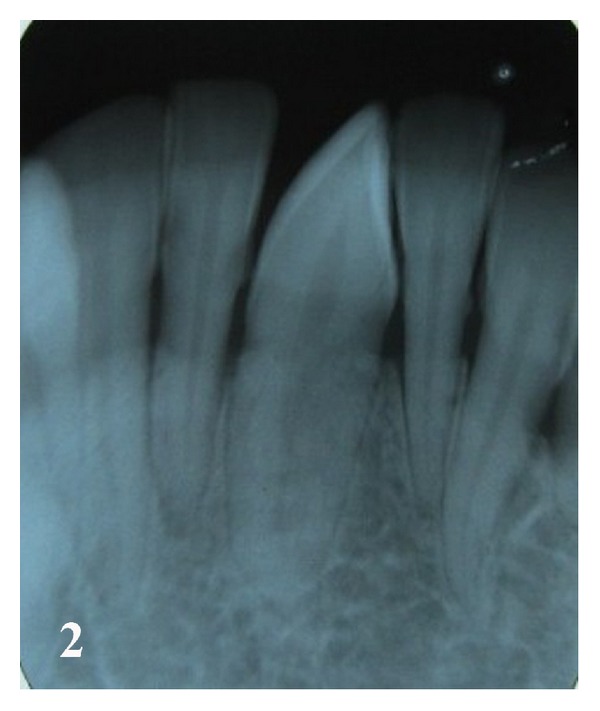
Periapical radiograph showing transmigrated and erupted lower left canine in the midline.

A 30-year-old female presented with the chief complaint of pain in upper left molar area. Intraoral examination showed a decayed upper first molar, and tooth simulating a mandibular left canine in the midline; the tooth was rotated and retained deciduous lower left canine. An intraoral periapical radiograph revealed erupted and transmigrated mandibular left canine in midline (Figure 2). The radiographic position of tooth was in accordance with type 5 transmigration pattern classified by Mupparapu [10]. The patient was informed of the condition and orthodontic alignment and reshaping of midline canine tooth with central incisor, and left lateral incisor with canine crown was decided for esthetic and functional reasons.

**Case 3**

**Figure 3:**
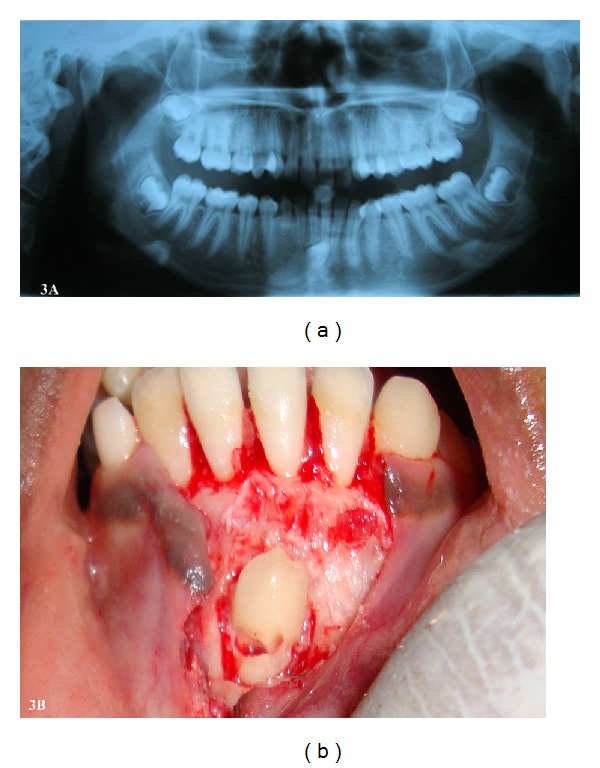
(a) Panoramic radiograph showing transmigrated lower right canine under the apices of the incisors. (b) Surgically exposed transmigrated lower right canine.

A 15-year-old female patient presented with the chief complaint of protruded upper front teeth. Intraoral examination revealed increased overjet, retained deciduous mandibular right canine, and clinically missing permanent right canine. Panoramic radiograph revealed transmigrated permanent right mandibular canine under the apices of the incisors. The radiographic position of tooth was in accordance with type 1 mandibular canine transmigration classified by Mupparapu [10] (Figure 3(a)). There was no pathologic finding associated with the transmigrated tooth. Surgical removal of the canine was done due to its unfavorable position (Figure 3(b)). Extraction of deciduous mandibular right canine and replacement with an endosseous implant and crown was advised.

**Case 4**

**Figure 4:**
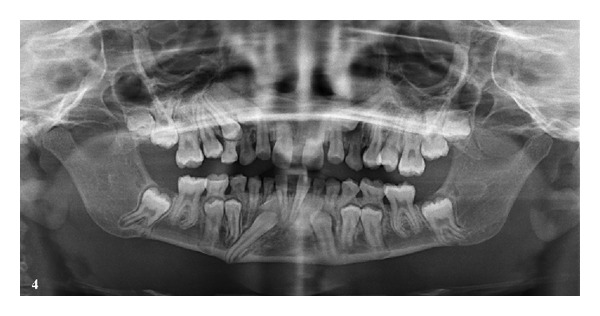
Panoramic radiograph showing an ectopically erupting mandibular right canine excessively tilted mesially and agenesis of lower lateral incisors.

A 12-year-old female patient presented with a complaint of spacing between upper front teeth. Intraoral examination revealed all deciduous canines intact and clinically nonpalpable all permanent canines, retained deciduous lower lateral incisors and clinically missing permanent lower lateral incisors. A Midline diastema was present in the upper arch. Panoramic radiograph revealed congenitally missing lower lateral incisors and an ectopically erupting mandibular right canine excessively tilted mesially (Figure 4). According to Howard's [11] criteria for canine transmigration, the axial inclination of mandibular right canine to midsagittal plane was 45°, and in addition it was associated with congenitally missing lower lateral incisors; hence, there is an increased tendency for the mandibular right canine to transmigrate. Therefore, preventive and interceptive treatment such as an extraction of the deciduous canine, surgical exposure of the ectopically erupting permanent canine followed by orthodontic treatment to pull the canine into the dental arch to its ideal position, was advised.

## 3. Discussion

Tarsitano et al. defined transmigration as a phenomenon in which an unerupted mandibular canine migrates, crossing the mandibular midline [12]. According to Joshi [3] migration of a tooth across the jaw midline without the influence of any pathological entity is called as transmigration. Javid [6] suggested that transmigration should be considered when half the length of the crown crosses the midline. 

In 2002, Mupparapu [10] classified mandibular canine transmigration depending on its path of deviation into five types. Type 1: canine positioned mesioangularly across the midline within the jaw bone, labial or lingual to anterior teeth, and the crown portion of the tooth crossing the midline (45.6%). Type 2: canine horizontally impacted near the inferior border of the mandible below the apices of the incisors (20%). Type 3: canine erupting either mesial or distal to the opposite canine (14%). Type 4: canine horizontally impacted near the inferior border of the mandible below the apices of either premolars or molars on the opposite side (17%). Type 5: canine positioned vertically in the midline (the long axis of the tooth crossing the midline) irrespective of eruption status (1.5%).

In the present study, the radiographic appearance of the transmigrated canine in Case 3 was in accordance with type 1. In Case 2, the radiographic position of the tooth was in accordance with type 5, where the transmigrated mandibular left canine was erupted in the midline.  Case 1 was not in accordance with any of the types described previously, as mandibular right canine was in a reverse oblique (110° to midsagittal plane) position, crossing the jaw midline, and piercing the lower border of the mandible at the level of the opposite canine. Hence, Case 1 is the first of its kind being reported in the literature. 

Pérez et al. [13] observed patients in the age ranging from 8 to 74 years old, with the mean age being 21.9 ± 13.4 years old. The greatest prevalence of transmigrated canines was found in the age group that ranges from 0 to 20 years, which accounted for 56.7% of the sample. The incidence of transmigrated canines is much higher in females than in males and there is no plausible reason why it occurs more often in women [3, 5, 7, 9, 12, 14].

The frequency of canine transmigration is more in the mandible than maxilla. It may be due to the larger distance between the root apices and lower border of mandible, and as the migratory canine is lodged in the symphyseal area, this area will grow larger to accommodate the migratory canine. Transmigration of maxillary canines is very rare, which may be due to the shorter distance between the roots of maxillary incisors and the floor of the nasal fossa and restriction of the path of tooth movement by the roots of adjacent teeth [14].

The etiology of this rare developmental anomaly is not yet known and there are many contributing factors to its occurrence. Heredity has been suggested as a causative factor. The most commonly accepted explanation is the abnormal displacement of the dental lamina in the embryonic life and noneruption of such canines. Noidine [15] emphasized that even a very small obstacle, such as a small root fragment or an odontoma, would be sufficient to divert a tooth from its normal path of eruption. Ando et al. [16] suggested that agenesis of permanent lateral incisors may result in deviated path of eruption and hence the transmigration. In our first case described previously, transmigrated mandibular canine was associated with congenitally missing central incisors which could be the cause for the transmigration. 

Al-Waheidi [17] suggested that transmigrated canines are usually associated with a cystic lesion and that the presence of a cyst at the crown of the canine may facilitate the migration process. Other authors, such as Joshi and Howard, did not report any associated cystic lesions with transmigration. However, the role of cystic lesions in the etiology of transmigration is difficult to determine. A cyst is an expansive lesion and is more likely to displace the tooth in any direction in the path of the least resistance. Among the transmigrated mandibular canine, cases reported here, 2 impacted and 1 erupted, were not found to be associated with any pathology.

### 3.1. Diagnosis

The presence of transmigration must be suspected in cases where lower permanent canine is clinically absent from the dental arch along with an abnormal retention of lower deciduous canine. The prolonged retention of the deciduous canine is quite often a reliable clue leading to the discovery of its impacted permanent successor. In this present paper retained deciduous canine was present in all four cases supporting the findings by Joshi [3] and Pérez et al. [13] in which 70.8% and 48% of the sample had retained deciduous canines, respectively.

The other clinical factors associated with canine transmigration are agenesis of lateral incisors, proclined lower incisors, and deviated dental midline [16]. When two or more of these characteristics are present and the patient is 10-to-13 year old, the patient should be evaluated radio-graphically for impaction and transmigration of permanent canine. Clinically, transmigration is usually asymptomatic. Joshi [3] reported that symptoms such as pain or oppression of mandibular nerve owing to the transmigration of canine in their patients were not observed.

It is possible in the routine checkup that the intraoral periapical radiograph may fail to reveal transmigrated canines. These canines may not be visible in periapical radiographs as some lie horizontally below the inferior alveolar canal or migrate toward the midline. This emphasizes the importance of a panoramic radiograph in diagnosis of canine transmigration. In Case 1, transmigration was detected only on panoramic radiograph. Since the advent of panoramic radiography, it has become very easy to detect a migratory tooth. 

Howard [11] observed the influence of the axial inclination of impacted canines on its migration. The author observed that those canines with axial inclination between 25° and 30° to the midsagittal plane represent a group of unerupted canines that are displaced but not migrating across the mandibular midline. Those impacted canines that are between 30° and 95° are a group that tends to cross the midline. An overlap appears to exist between 30° and 50°. When this angle exceeds 50°, crossing the midline becomes a rule. Joshi [3] reported the axial inclination to the midsagittal plane of migratory canine ranged from 45° to 95°. However, in our first case the described inclination of transmigrated tooth to midsagittal plane was 110^0^ which has not been reported in the literature.

Ando et al. [16] observed transmigration of canine in their patient for 6 years. During this time, the canine moved from its original position to a place near the mental foramen on the opposite side. They also suggested that the greatest amount of movement of transmigratory canine is more rapid before the formation of its root. However, Dhooria et al. [18] observed a fairly rapid movement even after completion of the root formation, about 3 to 4 mm in one of their patients during 1 year. Whereas in our first case, the mandibular right canine is transmigrated in a reverse oblique angulation, piercing the lower border of the mandible. A very thin plate of periosteal cortical bone is left covering the crown of the tooth. The tooth apex is closed and the patient is twenty-year old; if the tooth still has its inherent eruptive force, it may pierce the lower border of the mandible.

### 3.2. Treatment Options

Treatment considerations for transmigrated tooth depend on the stage of development, distance of migration, and position and angulation of the tooth when it is diagnosed. 

#### 3.2.1. Preventive and Interceptive Treatment

A small root fragment or an odontoma interferes with the normal path of the eruption of the tooth, hence, their removal would facilitate its eruption. Taguchi et al. [19] reported, after removal of the odontoma and surgical exposure an improvement in the position of the associated canines. Vichi and Franchi [20] suggested that an 8-to 9-year-old patient with an excessive mesial inclination of the unerupted mandibular permanent canine should be kept under critical observation with periodical panoramic radiographic examination. If the position of the unerupted mandibular canine is observed to progressively tilt more to the mesial, interceptive measures should be taken. The preventive and interceptive treatment includes extraction of the retained deciduous canine and surgical exposure of the impacted canine followed by orthodontic treatment. This emphasizes the importance of early diagnosis to correct this problem before the tooth migrates too far from its original location.

#### 3.2.2. Surgical Exposure and Orthodontic Treatment

The transmigrated canine, when detected early, can be surgically exposed and moved to its ideal position by using orthodontic forces. Wertz [21] reported the successful correction of three cases using orthodontic treatment, where lower canine was transmigrated labially. Kumar and associates [22] recently reported one case of successful eruption of transmigrated lower left canine after orthodontic traction, which was located with its crown below the apices of the right central incisor. However, if the crown of such a tooth migrates past the opposite incisor area or if the apex is seen to have migrated past the apex of the adjacent lateral incisor, it might be mechanically impossible to bring it into place [2].

Transplantation is another approach to save the transmigrated tooth. If the transmigrated canine is in favorable position for surgical removal in one piece asymptomatically and there is sufficient space with retained deciduous canine, transplantation may be undertaken. Timing of transplant is very important since a primary objective is to obtain maximum root length. The procedure is best performed when the root length of the tooth is between one-half and three-fourths complete for the reestablishment of blood supply. The prognosis is diminished as the root apex nears closure. The length of time from removal to reinsertion should be minimal; ideally, this is a nonstop relocation. Desiccation of the periodontal ligament can cause resorption, ankylosis, and failure [23]. Howard [11] transplanted a transmigrated canine when there was enough space, and retained deciduous canine was present to accommodate the tooth. Slagsvold and Bjercke autotransplanted the premolars with partly formed roots with successful results [24, 25].

Retrograde endodontic treatments have been performed simultaneous to transplantation with varying degrees of success; such treatment introduces a foreign substance into the site, possibly inducing an inflammatory reaction, but it greatly increases the length of the procedure and the time the transplant remains out of the mouth. Further, such manipulation will assuredly traumatize the root surface [23]. It would be more judicious to perform the root canal treatment after the periodontal ligament attachment has readapted if such treatment proves necessary. Verma et al. [26] reported a successful transplantation of transmigrated mandibular right canine (type 2 of Mupparapu) into the prepared socket of retained deciduous canine. After one year of followup they observed good periodontal condition with no gingival recession, but they also noted external root resorption at the distal side of the apex in the panoramic radiograph and axial section of CT scan.

#### 3.2.3. Surgical Removal

Wetz [21] stated that once the canine crown migrated past the adjacent lateral incisor root apex, orthodontically it is impossible to reposition the tooth to its ideal position and in cases where the transmigrated canine is associated with pathology or neurological symptoms, surgical removal is indicated. Bruszt [27] clearly showed that the transmigrated tooth maintains its nervous innervation from the side where the germ is formed. Joshi [3] suggested that it is important to anesthetize the nerve of the side to which the canine belongs, when the extraction is to be attempted under local anesthesia. However, if general anesthesia is to be used, this problem does not arise. He also suggested that the surgical removal of a transmigrated tooth should be done as far as possible through an intraoral approach. Nevertheless, if necessary, an extra oral approach can be used in extreme unusual cases of canine transmigration.

#### 3.2.4. Radiographic Monitoring

Impacted and transmigrated tooth can be left in place if it is symptomless and not associated with any pathology. In these patients, a series of periodic radiographs should be taken to check the status of the transmigrated tooth. A continuous worsening of position or development of cystic lesion and in case of severe root resorption of adjacent teeth, surgical removal is indicated [2].

#### 3.2.5. Transmigrated and Transpositioned Canine

In cases where the transmigrated canine is erupted and transpositioned in the dental arch, the tooth can be orthodontically aligned and recontoured accordingly. Trakyali et al. [28] reported the management of a transmigrated lower right canine, which was erupted between the left central and lateral incisors. The crown was recontoured to simulate a lateral incisor, and an acceptable aesthetic and functional outcome was gained. 

**Figure 5:**
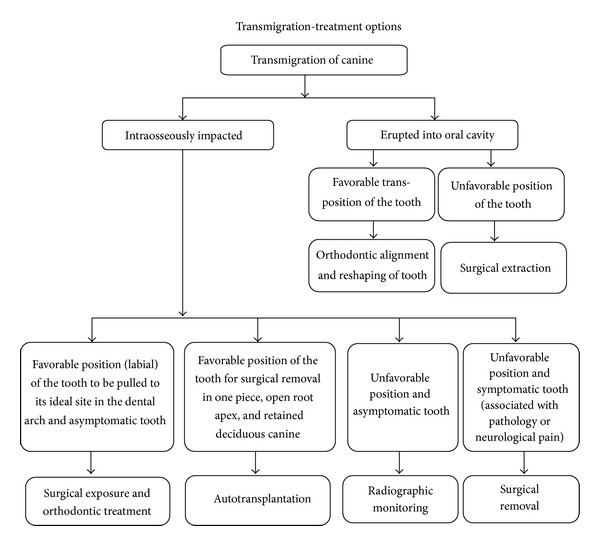
Flowchart depicting the various treatment options to aid in decision making for the management of canine transmigration.

A flowchart is provided which summarizes the various treatment options mentioned previously to guide in the decision making for management of canine transmigration (Figure 5).

## 4. Conclusion

With early radiographic detection and timely interception with surgical and orthodontic treatment, mandibular canines which have an increased tendency towards transmigration can be guided to erupt to its ideal position in the dental arch. However, if transmigration is detected at a later stage, autotransplantation is a better treatment option. Future studies may lead to a better understanding of this complex natured developmental anomaly leading to improvement in diagnosis and treatment.
